# Mucosal Margin Shrinkage in Oral Cavity Cancer: A Systematic Review

**DOI:** 10.1002/ohn.70244

**Published:** 2026-04-16

**Authors:** Sindhura Sridhar, Daniel P. Larson, Michael C. Topf

**Affiliations:** ^1^ Department of Otolaryngology–Head and Neck Surgery Vanderbilt University Medical Center Nashville Tennessee USA; ^2^ Vanderbilt University School of Medicine Nashville Tennessee USA

**Keywords:** margins, mucosal shrinkage, oral cavity cancer, systematic review

## Abstract

**Objective:**

Tissue shrinkage can make planning of surgical margins challenging. While shrinkage of resected oral cavity mucosa is well‐described, current data quantifying these effects are variable. The aim of this systematic review was to summarize current evidence on margin shrinkage in oral cavity cancer after resection and formalin fixation.

**Data Sources:**

PubMed, Embase, Web of Science, and Cochrane Database of Systematic Reviews.

**Review Methods:**

A systematic review was performed according to PRISMA guidelines to identify all published reports describing margin shrinkage in surgically resected oral cavity cancer. Only prospective cohort and cross‐sectional studies were included. Non‐English language studies, studies on non‐human subjects, and studies describing cancer of sites other than the oral cavity were excluded.

**Results:**

Ten studies met inclusion criteria, accounting for 352 patients who underwent resection of oral cavity cancer. Mean percent margin shrinkage from preresection to postresection ranged from 14.9% to 23.9%, from postresection to postfixation ranged from 3.7% to 32.9%, and from preresection to postfixation ranged from 11.3% to 59.0%. In studies reporting preresection, postresection, and postfixation margins (4 studies), preresection to postresection shrinkage was significantly larger than postresection to postfixation shrinkage. There was conflicting evidence on shrinkage by T stage and tumor subsite. There was no association between margin shrinkage and other clinicopathologic factors such as age, sex, or other adverse pathological factors.

**Conclusion:**

Oral cavity tissue undergoes shrinkage both after surgical resection and after fixation with formalin, with the most significant shrinkage occurring immediately after resection. Tissue shrinkage should be an important consideration in the planning of surgical margins.

Surgical margin status is an important prognostic factor in oral cavity cancer. Negative margins, typically defined as the resection edge greater than 5 mm from the closest invasive tumor, are associated with lower local recurrence‐free survival (LRFS) and improved overall survival (OS) rates compared to positive or close margins.^1^, ^2^ Current National Comprehensive Cancer Network (NCCN) guidelines defined adequate resection as enough clearance from gross tumor to obtain clear frozen section and permanent margins.^3^ However, attaining wide resection margins can be challenging due to anatomic limitations of the oral cavity and pharynx, aggressive tumor biology, and balancing resection of normal tissue and quality‐of‐life functions including swallowing, voicing, and oral competence.^4^

Achieving a clear 5 mm margin is additionally complicated by tissue shrinkage after surgical resection and formalin fixation. Tissue shrinkage results in discrepancy between margins measured in situ and final histopathological margins.^5^ Although microscopic tumor invasion or histopathological artifact can contribute to closer‐than‐expected margins, tissue shrinkage is often the primary underlying source of these discrepancies.^6^ As a result, head and neck surgical oncologists must resect a wider cuff of normal tissue around palpable tumor to approach the desired margin distance histologically.^4^ In addition, deformation of the specimen and resection bed can create challenges in re‐approximating specimen and margin orientation when re‐resection of positive or close margins is needed.

While the tissue shrinkage phenomenon is well‐known anecdotally, there is limited data quantifying its effects on surgical margins. Therefore, the objective of this study is to characterize patterns of mucosal deformation in the oral cavity after surgical resection and estimate the amount that shrinkage can affect margin measurement in oncologic surgery. In this systematic review, we summarize current evidence on margin shrinkage in oral cavity cancer after resection and formalin fixation.

## Methods

### Data Collection and Selection

This study was conducted according to the Preferred Reporting Items for Systematic Review and Meta‐Analyses (PRISMA) guidelines.^7^ PubMed (National Library of Medicine‐National Institutes of Health), Embase (Elsevier), Web of Science (Clarivate), and Cochrane Database of Systematic Reviews (Wiley) were searched from inception to October 2024. The search strategy was crafted with the assistance of a medical librarian to find studies reporting postresection shrinkage of surgical margins using the medical subject headings (MeSH) and the following keywords: oral cavity; oral cancer; surgical margins; surgical resection; discrepancy; shrinkage. The complete PubMed search strategy is available in Supplement S1, available online. All articles from the search were exported into Covidence (Veritas Health Information Ltd.). Title, abstract, and full‐text screening were performed by 2 independent reviewers (SS and DPL).

### Selection Criteria

This study aimed to identify all published reports describing margin shrinkage in surgically resected oral cavity cancer. Only prospective cohort and cross‐sectional studies were considered. Studies providing quantitative margin measurements of oral cavity tumor specimens after surgical resection or fixation with formalin were included. Studies describing changes in specimen shrinkage, tumor size, specimen area, or specimen volume without reporting changes in margin size were excluded. Non‐English language studies, studies on nonhuman subjects, studies on subjects with no cancer, and studies describing cancer of sites other than the oral cavity were excluded.

### Data Extraction

Data extraction and quality assessment were performed by 2 independent authors (SS and DPL). Any disagreements were resolved by a third reviewer (MCT). Data extracted included study characteristics, such as first author, year of publication, article title, and journal; patient demographics; measurement timepoints (preresection, postresection, and/or postfixation with formalin); mean individual measurements for each timepoint or mean percent change in size across timepoints; and tumor subsite and T stage if reported.

### Quality Assessment

All included studies underwent rigorous risk of bias (ROB) assessment using the National Institutes for Health (NIH) Quality Assessment Tool for Observational Cohort and Cross‐Sectional Studies (Supplement S2, available online).^8^ A score of at least 7.5 was required for inclusion in the study.

## Results

### Study Demographics

**Figure 1:**
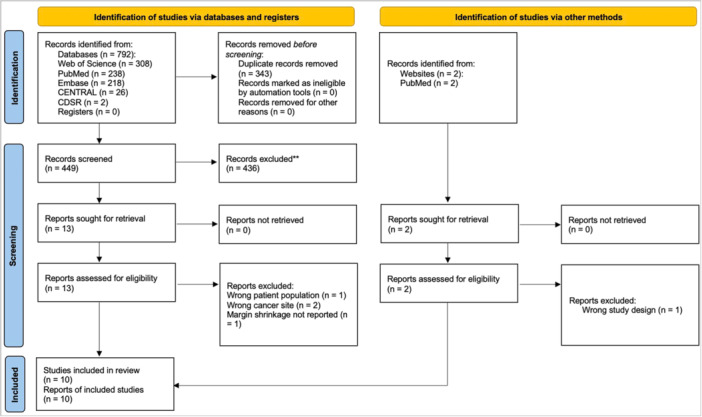
PRISMA diagram.

We initially identified 449 studies. These were narrowed to 13 studies on title and abstract screening based on exclusion criteria, and then to 9 studies which were included for analysis after full‐text review.^6^, ^9^, ^10^, ^11^, ^12^, ^13^, ^14^, ^15^, ^16^ Figure 1 demonstrates the PRISMA diagram. In addition to the predefined database search, 2 additional studies published after the conclusion of initial analysis were identified through a targeted search for relevant literature conducted during manuscript preparation.^17^, ^18^ Of these, 1 study met inclusion criteria and was incorporated into data synthesis.^17^

The 10 included studies accounted for 352 patients. All studies were prospective observational studies, involving a variety of cohort sizes, tumor subsites, T stages, resecting instruments, and measurement points (Table 1). Resecting instruments were reported in 5 studies. Three studies reported using electrocautery, 1 study used harmonic scalpel for glossectomies and monopolar electrocautery for buccal mucosa, and 1 study used a scalpel for mucosal and skin incisions with electrocautery to complete the excision; no study reported power settings of the instrument. Changes in margin size were measured using different methods in each study. All studies delineated in situ margins using sutures or a marking pen. Six studies delineated 1 cm or greater margins, whereas 4 studies measured in situ margins that were determined by the resecting surgeon. After resection, studies performed measurements at postresection, postfixation with formalin, and/or posthistopathological processing timepoints, and calculated mean margin shrinkage between timepoints. Only 4 studies reported changes between preresection and postresection, postresection and postfixation, and preresection and postfixation timepoints; the other 6 studies reported mean percent change in margin size between at least 2 timepoints. Studies did not consistently distinguish between postfixation and postprocessing timepoints, and final histopathological margin measurements were used to evaluate the role of formalin in margin shrinkage in multiple studies. In addition, the time or volume amount of formalin fixation was not consistently documented, and in studies that reported this time, fixation ranged from a minimum of 1.6 hours to a maximum of 48 hours after resection. Due to the heterogeneity of study designs, we did not conduct a meta‐analysis.

**Table 1:** Study Demographics

Author	Year	Subjects	Mean age	Tumor subsite	T Stage
Mistry et al	2005	27	47.6 (36‐31)	Tongue, buccal mucosa	T1, T2, T3, T4
Cheng et al	2008	41	67 (35‐95)	Tongue, buccal mucosa, mandibular/maxillary ridge, hard palate, retromolar trigone	T1, T2, T3, T4
Egemen et al	2014	21	71.1 (47‐92)	Lip	T1, T2, T3
El‐Fol et al	2015	61	51.6 (35‐69)	Tongue, buccal mucosal, alveolar margin, retromolar trigone, floor of mouth	T1, T2, T3, T4
Mohiyuddin et al	2016	52	55.2 (45‐65)	Buccal mucosa	T2, T3, T4
Umstattd et al	2017	19	Median age: 71 (33‐84)	Tongue, alveolar/gingival, floor of mouth, retromolar trigone	Did not specify
Pangare et al	2017	15	58.2 (55‐75)	Gingivobuccal sulcus	T3, T4
Burns et al	2021	6	65.5 (51‐91)	Oral tongue, buccal mucosa	T1, T2, T3
Kshithi et al	2022	50	Did not specify	Tongue, buccal mucosa, lip	T1, T2, T3, T4
Vaidya et al	2025	44	Median age: 54.5 (32‐92)	Tongue, buccal mucosa, gingivobuccal sulcus, lip, alveolus, floor of mouth, retromolar trigone	T1, T2, T3, T4a

### Margin Shrinkage After Resection and Formalin Fixation

Eight studies reported mean margin shrinkage for the overall cohort. Egemen et al reported mean margin shrinkage for the left and right margins for each specimen,^14^ and Pangare et al reported mean changes in anterior, posterior, medial, and lateral margins for each specimen.^11^ Since the objective of this systematic review was to estimate mucosal deformation in oral cavity cancer, the mean shrinkage for individual margin locations from these 2 studies was included in data synthesis. Table 2 summarizes the key findings from each study.

**Table 2:** Mean Percent Margin Shrinkage

Mean percent margin shrinkage						
Author	Preresection to Postresection	Postresection to Postfixation	Preresection to Postfixation	Tumor subsite	T stage	Miscellaneous
Mistry et al	22.7%^a^			Tongue: 23.5%; Buccal: 21.2%	T1/T2: 25.6%; T3/T4: 9.2%^a^	No difference by age or sex
Cheng et al			59.0%	Buccal mucosa/mandibular alveolar ridge/retromolar trigone: 71.9%; Maxillary alveolar ridge/palate: 53.3%; Tongue: 42.1%^a^	T1/T2: 51.5%; T3/T4: 75%	
Egemen et al	22.0% (R)^a^; 18.0% (L)^a^	32.7% (R)^a^; 27.7% (L)^a^	47.5% (R)^a^; 41.0% (L)^a^			
El‐Fol et al			Did not report percent shrinkage; reported as significant^a^	Buccal mucosa: 47.6%; FOM: 4.8%; Mandibular alveolus: 9.5%; Retromolar trigone: 4.8%; Tongue: 33.3%^a^		
Mohiyuddin et al		25.0%^a^				
Umstattd et al	14.9%^a^	3.7%^a^	11.9%^a^			
Pangare et al		18.7% (A)^a^; 14.9% (P)^a^; 23.6% (M)^a^; 23.9% (L)^a^				No difference by age, sex, or tumor size
Burns et al	19.7%^a^	12.7%	26.0%^a^			
Kshithi et al		25.6%^a^		Buccal: 24.75%; Tongue: 27.73%; Lip: 24.48%		
Vaidya et al	15.31%^a^	7.3% (postresection to postfixation)^a^; 10.8% (postfixation to histopathological processing)^a^	33.4%^a^	Did not report percent shrinkage; reported as not significant	Did not report percent shrinkage; reported as not significant	No difference by age, sex, N‐stage, LVI, PNI, WPOI, grade

^a^ Indicates that result was statistically significant.

Of the included studies, 5 reported a decrease in mean margin size from preresection to postresection,^9^, ^13^, ^14^, ^15^, ^17^ 7 reported decrease in mean margin size from postresection to postfixation with formalin,^9^, ^10^, ^11^, ^12^, ^14^, ^15^, ^17^ and 5 reported decrease in mean margin size from preresection to postfixation with formalin.^6^, ^9^, ^14^, ^15^, ^17^ Mean percent margin shrinkage from preresection to postresection ranged from 14.9% to 23.9%. Postresection to postfixation margin shrinkage ranged from 3.7% to 32.7%. Finally, total preresection to postfixation shrinkage ranged from 11.3% to 59.0%. All studies found a statistically significant difference between preresection and postresection or postfixation margins; however, only 5 of 7 studies found a statistically significant difference between postresection and postfixation margins.^9^, ^10^, ^11^, ^12^, ^14^, ^15^, ^17^ In addition, in the studies reporting measurements at all 3 timepoints, shrinkage of margins from preresection to postresection was greater than shrinkage between postresection to postfixation in 3 out of 4 studies.^9^, ^14^, ^15^, ^17^

### Clinicopathologic Factors and Margin Shrinkage

The 10 studies included specimens comprising all oral cavity subsites, including buccal mucosa, oral tongue, floor of mouth, mandibular/maxillary alveolar ridge, gingivobuccal sulcus, retromolar trigone, hard palate, and lip. Four studies reported shrinkage by tumor subsite,^6^, ^12^, ^13^, ^16^ and an additional 2 studies evaluated margins from only 1 tumor subsite.^11^, ^14^

In studies that conducted comparative analysis of margin shrinkage by tumor subsite, 2 studies found significant differences between tumor subsites, whereas 2 studies found no discrepancy in margin shrinkage by tumor subsite. Cheng et al and El‐Fol et al found greater discrepancy in shrinkage of buccal mucosa (and contiguous structures) compared to oral tongue mucosa.^6^, ^16^ Both Mistry et al and Kshithi et al found no significant differences between buccal and oral tongue mucosa.^12^, ^13^ Egemen et al only included specimens from lip cancer resections, reporting a mean shrinkage of 41.0% to 47.5% from preresection to postfixation.^14^ Pangare et al only included specimens from resection of tumors from the gingivobuccal sulcus; the reported change in margin size from postresection to postfixation was 20.3%.^11^

The 10 studies included specimens comprising all pathological T stages, from T1 to T4. Only 2 studies reported differences in margin shrinkage by T stage, with conflicting results. Cheng et al found that T3/T4 tumors have significantly larger shrinkage than T1/T2 tumors (75% vs 51.48%, *P* = .0264),^6^ whereas Mistry et al found that T1/T2 tumors have greater shrinkage than T3/T4 tumors (25.6% vs 9.2%, *P* < .011).^13^

A select number of the included studies have investigated the role of patient age, sex, and other clinicopathological factors in mucosal margin shrinkage. However, no studies found any associations between other clinicopathologic factors and the degree of margin shrinkage.

## Discussion

Tissue shrinkage is a well‐known yet understudied phenomenon that can greatly affect the outcome of surgical margins. In this study, we systematically reviewed current literature describing margin shrinkage in oral cavity cancer. There is significant variability in the reported degree of margin shrinkage, although shrinkage is consistently observed after surgical resection and formalin fixation regardless of location and severity of disease. Planned margins can shrink between 11.3% and 59.0% in between measurement in situ and histopathological examination. Margin shrinkage is most immediately observable after surgical resection, with a 14.9% to 23.9% decrease in size between specimen margins measured in situ and after excision. An additional 3.7% to 32.9% of shrinkage can occur because of formalin fixation. The role of tumor subsite and T stage in margin shrinkage is less clear and understudied, with conflicting results reported in current literature. Finally, other clinical factors such as patient age or sex, and pathologic factors such as perineural invasion (PNI) and lymphovascular invasion (LVI) do not seem to affect margin shrinkage, although current literature contains limited supporting data.

The patterns of margin shrinkage captured in this study are consistent with those described in other cancers. Prior studies demonstrate between 9.5% and 45% margin shrinkage in cutaneous cancers^19^, ^20^, ^21^, ^22^ and between 40% and 54% margin shrinkage in digestive tract cancers between in vivo and ex vivo measurements.^23^, ^24^ Studies examining shrinkage after formalin fixation have found between 10.5% and 17% margin contraction in colorectal cancer,^24^, ^25^ 34% margin contraction in breast cancer,^26^ 32% to 39% in esophageal cancer,^23^ and 25% to 39% in cutaneous cancers.^22^ Across tumor types, the greatest percentage of margin shrinkage occurred immediately after tumor excision with additional shrinkage occurring after formalin fixation, mirroring deformation behavior in oral mucosa and confirming the importance of accounting for tissue contractility when planning oncologic resection.

Surgical resection releases tissue from the underlying muscle and surrounding structures, resulting in immediately observable shrinkage of the specimen margins and expansion of the tumor bed. Oral cavity mucosa is highly organized and contractile, and disruption of the tissue organization causes unopposed contractility of the tissue at the resection margin.^5^ Neutral‐buffered formalin contains 4% formaldehyde, which forms crosslinks between proteins or proteins and nucleic acids and coordinate (covalent) bonds with calcium ions over the course of 24 to 48 hours.^27^ These crosslinks and coordinate bonds have been theorized to alter molecular architecture, resulting in further observed shrinkage. Unsurprisingly, all studies in this systematic review consistently observed shrinkage at each step between preresection margin measurement and histopathological examination. The degree of shrinkage was always significant after resection, and largely significant after formalin fixation. There were 2 studies that did not demonstrate a significant degree of shrinkage after formalin fixation. In the study by Umstattd et al who reported a 3.7% mean margin shrinkage after formalin fixation, reported specimen time in formalin was between 1.6 and 31.7 hours with a mean of 7.7 hours^15^; Burns et al reported 12.7% mean margin shrinkage after 1 to 2 days of formalin fixation for all specimens.^9^ The time‐dependent nature of formalin fixation likely contributes to the variability of observed effects compared to resection alone.

The degree of tissue deformation is also affected by the type and power settings of the resecting instrument. Warshavsky et al demonstrated in rat models that the same excision of oral cavity mucosa performed with cold steel or monopolar electrocautery at 20 and 30 W cut modes showed significant differences in shrinkage, with the greatest specimen contraction observed with the monopolar electrocautery at the highest power setting.^28^ Electrocautery delivers thermal damage to the tissue resulting in additional contraction after natural tissue shrinkage; as stronger power settings are applied, increasing thermal injury results in greater contraction.^28^ Of the studies included in this systematic review, 3 used electrocautery only, 1 used cold steel and electrocautery, and 1 used harmonic scalpel and monopolar electrocautery, with instrumentation unknown for the other 5 studies and electrocautery power settings unknown for all studies. Although a statistically significant degree of shrinkage was consistently observed between preresection and postresection, the variety of instruments and possible power settings applied across studies likely contributes to the wide range of shrinkage observed across studies.

This systematic review found conflicting evidence as to the role of tumor subsite in margin shrinkage. Oral mucosa is diverse in function, and deformation occurs relative to the structure and organization of the tissue. Masticatory mucosa, such as gingiva, and lining mucosa, such as buccal and lip mucosa, must accommodate and withstand the forces of mouth opening, mastication, and swallowing, which exert significant mechanical stresses on the tissue, and therefore have a highly organized collagen structure and increased elastin content.^29^, ^30^ In comparison, the oral tongue is responsible for more specialized functions, is more muscular, and in turn, has decreased collagen organization.^31^ The landmark study by Johnson et al describing margin shrinkage in the oral cavity demonstrated this difference in canines: labiobuccal mucosal margins shrunk by 38.3% after resection and by an additional 10.5% after formalin fixation, compared to 24.8% postresection shrinkage and an additional 7.6% after formalin fixation in oral tongue mucosa.^5^ The studies included in our systematic review are limited by small sample sizes and heterogeneity of study design; however, given the variability in function and structure of oral cavity mucosa, deformation behavior is likely unique to subsite. Cheng et al postulate that differences in contractility of tissue, susceptibility to invasion, or biological heterogeneity of tumors by location within the oral cavity may further contribute to differences in margin shrinkage by subsite.^6^ When resecting within the oral cavity, an appreciation of the contractility of the tissue at the proposed area of excision is important in planning an appropriately wide resection margin.

The role of T stage in affecting margin shrinkage is similarly unclear. Mistry et al, who observed greater shrinkage in T1/T2 tumors compared to T3/T4 tumors, hypothesized that margins in T1/T2 tumors are likely to be more elastic and therefore shrink more compared to T3/T4 tumors.^13^ Cheng et al, who reported greater shrinkage in late‐stage tumors, conjectured that greater microscopic invasiveness of T3/T4 tumors could cause increased disruption of tissue architecture.^6^ Multiple studies have demonstrated that increased microscopic invasiveness correlates with increased likelihood of close or positive margins,^1^, ^32^, ^33^ and therefore closer margins caused by shrinkage are a greater concern. Although microscopic invasiveness often cannot be established prior to histopathological examination, gross correlators of aggressive tumor behavior such as greater T stage, endophytic tumor growth, or clinical nodal disease, may need to be excised with wider margins to account for both potential margin shrinkage and microscopic tumor invasion.

One consideration to note is that the patterns of deformation described in this systematic review may not apply to previously irradiated tissue. Of the included studies, 6 excluded previously treated or radiated patients, 3 did not explicitly state whether previously treated patients were excluded, and 1 study included 2 patients with previous radiation to the head and neck. Radiation alters intrinsic tissue properties and affects collagen production, resulting in decreased elasticity and fibrosis over time.^34^, ^35^ Further study is needed to appropriately characterize margin shrinkage in the salvage surgery setting.

Tissue deformation affects several aspects of intraoperative decision‐making, from surgical planning to margin evaluation, and re‐resection. Current NCCN guidelines recommend resecting enough grossly normal tissue surrounding palpable tumor to achieve negative margins on frozen and permanent section, typically 10‐15 mm,^3^ to account for margin shrinkage and other factors that may result in closer than expected microscopic margins. However, given the anatomic limitations of the oral cavity, this is not always possible. Understanding patterns of tissue shrinkage is important for surgical planning and maximizing clear histopathological margins.

### Limitations

There are several limitations to this study. Most importantly, this systematic review comprises a limited number of studies with heterogeneous study designs and small sample sizes. This limited definitive quantitative assessment of deformation after resection and formalin fixation. This also limited assessment of margin shrinkage related to clinicopathologic factors which have a known effect on margin status. In addition, there were small differences in study design which may have confounded evaluation of margin shrinkage. For example, Umstattd et al did not exclude patients with prior treatment of their cancer.^15^ Mohiyuddin et al included patients with verrucous squamous cell carcinoma (SCC).^10^ These study differences introduce factors that could affect tissue contractility; prior treatment reduces tissue elasticity and SCC variants may have different tumor behavior, affecting margins differently. Future study with greater sample sizes and more robust study design is needed to clarify and further define patterns of tissue deformation in oral cavity mucosa.

## Conclusion

In conclusion, oral cavity margins undergo shrinkage both after surgical resection and after fixation with formalin. The most significant shrinkage occurs immediately after resection, with a mean of 14.9% to 23.9% shrinkage. Further study is needed to clarify and further define patterns of mucosal deformation in oral cavity mucosa by T stage and tumor subsite.

## Author Contributions

**Sindhura Sridhar**, study design, abstract and article review, data extraction and analysis, manuscript writing and revision; **Daniel P. Larson**, abstract and article review, data extraction, manuscript revision; **Michael C. Topf**, supervision, study design, manuscript revision.

## Disclosures

### Competing interests

None.

### Funding source

This work was supported by a National Cancer Institute (NCI) K08 Career Development Award − 5K08CA293255‐02.

## Supporting information

Supplement 1. PubMed Search Strategy – Keywords and Medical Subject Headings (MeSH) terms used to query PubMed.

Supplement 2. National Institutes of Health Quality Assessment Tool for Observational Cohort and Cross‐Sectional Studies – Quality assessment tool and completed risk of bias assessment for all included studies. A score of at least 7.5 was required for inclusion in the study.
