# Supplementary material for: Mucosal Margin Shrinkage in Oral Cavity Cancer: A Systematic Review
Source: Otolaryngol Head Neck Surg. 2026 Apr 16;175(1):31–8. doi: 10.1002/ohn.70244 (PMC13327500; doi:10.1002/ohn.70244)
Supplement: Supplementary file 1 — Supplement 1. PubMed Search Strategy – Keywords and Medical Subject Headings (MeSH) terms used to query PubMed. [file OHN-175-31-s001.docx]

**Supplement 1. PubMed Search Strategy**

("Mouth Neoplasms"[Mesh] OR "Squamous Cell Carcinoma of Head and Neck"[Mesh] OR ((mouth[tiab] OR lip[tiab] OR oral[tiab] OR intraoral[tiab] OR tongue[tiab] OR head[tiab] OR neck[tiab] OR gingiva*[tiab] OR palat*[tiab] OR buccal[tiab] OR retromolar trigone[tiab]) AND (cancer[tiab] OR neoplasm*[tiab] OR carcinoma*[tiab] OR tumor*[tiab] OR tumour*[tiab]))) AND ("Margins of Excision"[Mesh] OR resection[tiab] OR post-resection[tiab] OR margin*[tiab]) AND (shrinkage*[tiab] OR discrepanc*[tiab] OR "difference measurements"[Title/Abstract:~2])
